# Structural and Functional Hierarchy in Photosynthetic Energy Conversion—from Molecules to Nanostructures

**DOI:** 10.1186/s11671-015-1173-z

**Published:** 2015-12-01

**Authors:** Tibor Szabó, Melinda Magyar, Kata Hajdu, Márta Dorogi, Emil Nyerki, Tünde Tóth, Mónika Lingvay, Győző Garab, Klára Hernádi, László Nagy

**Affiliations:** Department of Medical Physics and Informatics, University of Szeged, Rerrich B. tér 1., H-6721 Szeged, Hungary; Biological Research Center, Hungarian Academy of Sciences, Temesvari krt.62, H-6726 Szeged, Hungary; Biophotonics R&D Ltd., Temesvari krt.62, H-6726 Szeged, Hungary; Department of Applied and Environmental Chemistry, University of Szeged, Szeged, Hungary

**Keywords:** Photosynthesis, Bio-composites, Nano-composites, Bioelectronics

## Abstract

Basic principles of structural and functional requirements of photosynthetic energy conversion in hierarchically organized machineries are reviewed. Blueprints of photosynthesis, the energetic basis of virtually all life on Earth, can serve the basis for constructing artificial light energy-converting molecular devices. In photosynthetic organisms, the conversion of light energy into chemical energy takes places in highly organized fine-tunable systems with structural and functional hierarchy. The incident photons are absorbed by light-harvesting complexes, which funnel the excitation energy into reaction centre (RC) protein complexes containing redox-active chlorophyll molecules; the primary charge separations in the RCs are followed by vectorial transport of charges (electrons and protons) in the photosynthetic membrane. RCs possess properties that make their use in solar energy-converting and integrated optoelectronic systems feasible. Therefore, there is a large interest in many laboratories and in the industry toward their use in molecular devices. RCs have been bound to different carrier matrices, with their photophysical and photochemical activities largely retained in the nano-systems and with electronic connection to conducting surfaces. We show examples of RCs bound to carbon-based materials (functionalized and non-functionalized single- and multiwalled carbon nanotubes), transitional metal oxides (ITO) and conducting polymers and porous silicon and characterize their photochemical activities. Recently, we adapted several physical and chemical methods for binding RCs to different nanomaterials. It is generally found that the P^+^(Q_A_Q_B_)^−^ charge pair, which is formed after single saturating light excitation is stabilized after the attachment of the RCs to the nanostructures, which is followed by slow reorganization of the protein structure. Measuring the electric conductivity in a direct contact mode or in electrochemical cell indicates that there is an electronic interaction between the protein and the inorganic carrier matrices. This can be a basis of sensing element of bio-hybrid device for biosensor and/or optoelectronic applications.

## Review

### Introduction

Photosynthesis, the conversion of light energy into chemical energy by living organisms, is the energetic basis of virtually all life on Earth; also, fossil fuels are energy deposits of photosynthesis of past million years. The photosynthetic utilization of light energy requires a machinery organized in a structural and functional hierarchy from molecules through protein complexes to entire organisms [1]. One of the major goals of photosynthesis research, conducted worldwide in academic laboratories and industrial institutes, is to find biomimetic and biotechnological applications and technologies of utilization of the environmentally safe and essentially inexhaustible solar energy. One interesting concept is green synthesis of nanoparticles for diverse application in almost all fields of medicine, agriculture and technology reviewed by Husen and Siddiqi [2]. In addition, new generations of light-harvesting and photoactive intelligent materials are also of high interest [3–10]. Bio-nano-composite materials are considered the materials for the future [11, 12]. Biological systems offer inherently good examples for phenomena in nanotechnology, since the length scales of functional molecular assemblies, such as protein complexes and membrane fragments, fall in the 5–10-nm range. In the following sections, examples of nanotechnological applications of photosynthetic systems will be presented, with our attention focused mainly on systems containing purple bacterial reaction centres.

### The Basic Molecules of the Photosynthetic Energy Conversion Are the Chlorophylls

**Fig. 1:**
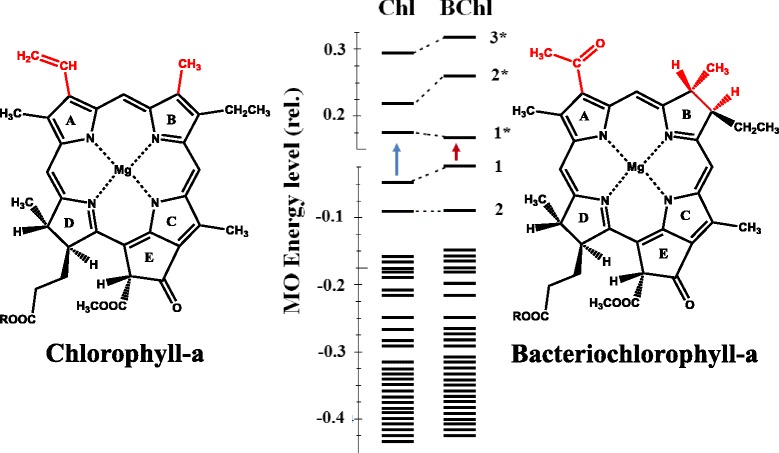
The molecular structures (*left and right*) and energy levels (ground state-binding and excited state-unbinding orbitals (labelled by *asterisks*), in the *middle*) of chlorophyll-a and bacteriochlorophyll-a molecules. *Arrows* indicate the electronic transitions between the highest occupied and lowest unoccupied molecular orbitals, HOMO and LUMO, respectively. The basic differences between the two molecular structures are indicated by *red characters*

**Fig. 2:**
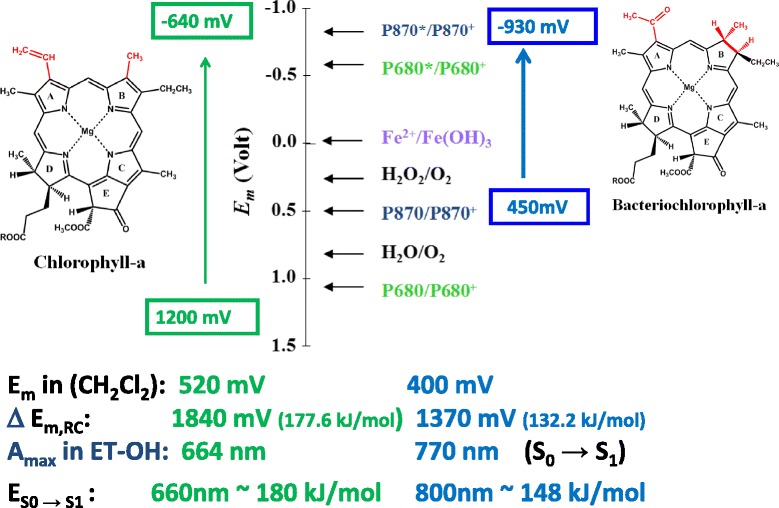
Comparison of basic photochemical and photophysical characteristics of chlorophyll-a and bacteriochlorophyll-a. The redox middle potential, *E*_m_, in dichloromethane and the wavelength of the absorption maxima, *A*_max_, in the red and near infrared are also indicated. For comparison, the *E*_m_ of several characteristic redox couples are also indicated. Redox couples: *P680/P680* ^*+*^: ground state PS-II primary donor; *H*_*2*_ *O/O*_*2*_: water/oxygen; *P870/P870* ^*+*^: ground state bacterial RC primary donor; *H*_*2*_ *O*_*2*_ */O*_*2*_: hydrogen peroxide/oxygen; *P680* ^*^ */P680* ^*+*^: excited state PS-II primary donor; *P870* ^*^ */P870* ^*+*^: excited state purple bacterial RC primary donor

The photosynthetic energy conversion begins with the absorption of light by the photosynthetic pigments, most notably by chlorophyll molecules. The special biological and chemical functions of chlorophylls are determined by their molecular structures, containing highly delocalized conjugated molecular orbitals (Figs. 1 and 2). The energy difference between the highest occupied molecular orbital (HOMO) and lowest unoccupied molecular orbital (LUMO) is reflected in the spectroscopically measurable S_0_ → S_1_ transition which is about 660 nm for chlorophyll-a in organic solution (see Fig. 2). Chlorophyll-a is a redox-active pigment with a redox mid-potential of *E*_m_ ≈ 500 mV in organic solution. When the chlorophylls are bound to proteins (e.g. in the photosynthetic reaction centre (RC) protein, the site of primary photochemistry), the *E*_m_ is shifted to a more positive (oxidizing) value, *E*_m_ = 1200 mV. It is interesting to note that this is the most oxidizing redox system in living cells and fulfils the energetic requirements of water splitting; the *E*_m_ of water/oxygen system is *E*_m_ = 820 mV.

The importance of the molecular structure in determining the biological function can be understood when the properties of the chlorophylls are compared with those of bacteriochlorophylls, found in un-oxygenic photosynthetic prokaryotes. In bacteriochlorophyll, the conjugation of the electron shells is disrupted (Fig. 1). The lower degree of delocalization increases the energy level of HOMO and decreases, in turn, that of LUMO. This change in the energy difference is reflected by spectroscopic features. The S_0_ → S_1_ transition for bacteriochlorophyll-a corresponds to *λ* = 760 nm in organic solution (in contrast to *λ* = 660 nm for chlorophyll-a), and the oxidizing power is smaller, *E*_m_ = 400 mV (as opposed with 500 mV for chlorophyll-a) in organic solution. When bacteriochlorophylls form the special pair in the RC protein, *E*_m_ is shifted only moderately (from 400 to 450 mV); this is far too low with respect to the energetic requirement of water oxidation.

It is important to note that the difference in the *E*_m_ values between the excited and ground state couples of the primary donors are Δ*E*_m_ = 1840 mV (corresponding to a free energy difference, Δ*G* = 177.6 kJ/mol) and Δ*E*_m_ = 1370 mV (Δ*G* = 132.2 kJ/mol) for chlorophyll-a and bacteriochlorophyll-a, respectively. These values coincide with the S_0_ → S_1_ transitions in the red (*λ* = 660 nm (Δ*G* = 180 kJ/mol)) and near infrared *λ* = 800 nm (Δ*G* = 148.5 kJ/mol) for chlorophyll-a and bacteriochlorophyll-a, respectively. For summary, see Fig. 2.

### Organization of the RC Protein

The photosynthetic pigments (chlorophylls and bacteriochlorophylls, together with other molecules) are arranged in special pigment-protein complexes (light-harvesting antenna complexes and RC proteins) in which efficient light absorption, energy transfer, charge separation and stabilization are warranted. Upon excitation by light primary charge, separation takes place in the photosynthetic RCs, which are the most efficient light energy converter systems in nature [6, 13]. Although different types of RCs have been evolved in nature (photosystem I (PS-I) and photosystem II (PS-II) in plants, algae and cyanobacteria and type I and type II RCs in green and purple photosynthetic bacteria, respectively), they all perform very similar basic processes: pigment excitation upon the absorption of light, charge separation and stabilization, rearrangements of charges, and hydrogen bonding interactions within and around the RC proteins.

**Fig. 3:**
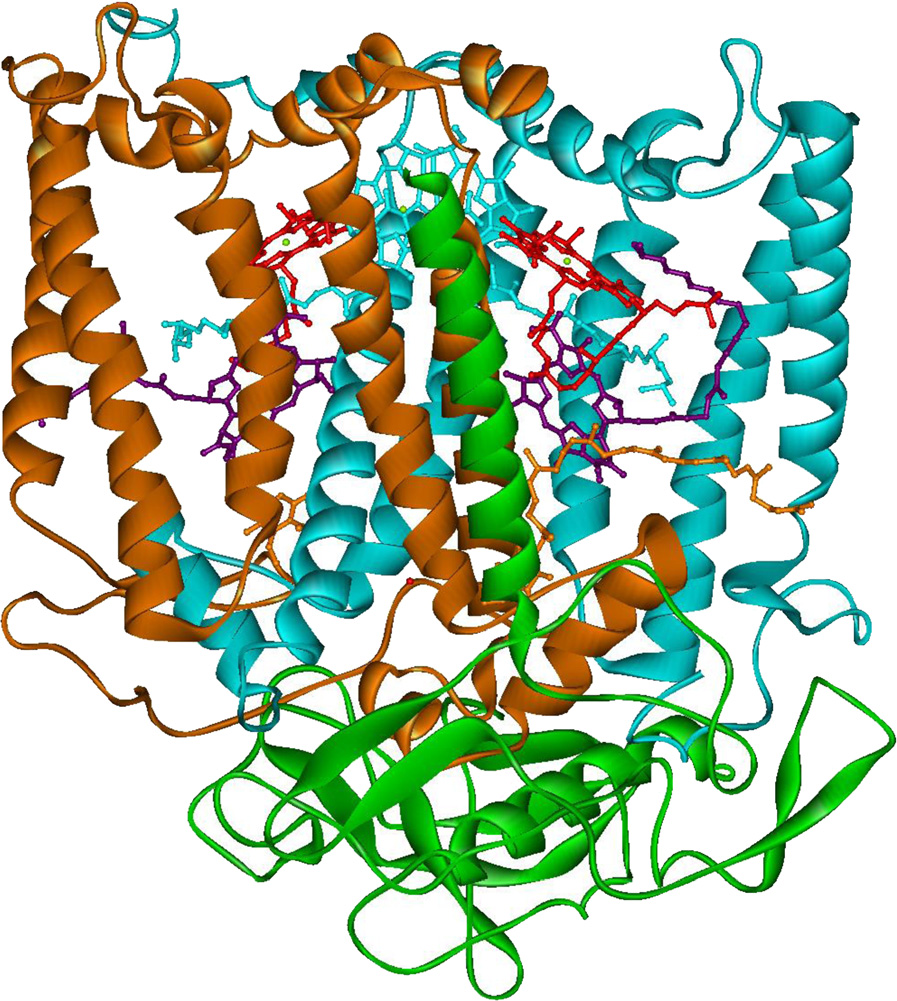
The structure of the RC protein of *Rhodobacter sphaeroides* R-26 (an anoxygenic purple bacterium). The secondary structures of the protein (H: *green*, L: *brown*, M: *blue*) subunits are shown by ribbons. The cofactors bound to the RC are also displayed; for further details, see Fig. 4. The molecular model of the reaction centre was calculated from the data of crystal structure downloaded from the Brookhaven Protein Data Bank (www.rcsb.org, code name: 1pst)

**Fig. 4:**
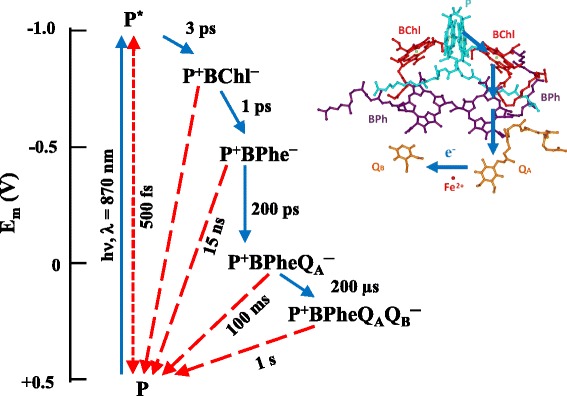
The lifetimes of the forward (*blue solid arrows*) and the backward recombination (*red dashed arrows*) electron transfer steps between the cofactors of the RC. *Insert* shows the arrangement of the cofactors and the pathway of the electron transfer after saturating single-turnover flash excitation. *P*: primary electron donor; *BChl*: bacteriochlorophyll; *BPhe*: bacteriopheophytin; *Q*_*A*_: primary quinone type electron acceptor; *Q*_*B*_: secondary quinone type electron acceptor

In purple bacteria, the absorption of light initiates a vectorial e^−^ transport in the RC—electrons are transferred from the specially organized chlorophyll or bacteriochlorophyll type primary e^−^ donor (P) through redox-active cofactor pigments to quinone (Q) acceptor molecules (for review, see, e.g. [14–16]). Finally, a pair of “+” and “−” centres (P^+^Q_A_^−^ or P^+^Q_B_^−^ for fully reconstituted RCs) is created after stabilization of the excited electron (Figs. 3 and 4). In vivo (or in artificial systems mimicking the in vivo conditions), the oxidized primary donor is reduced by an external electron donor, and the excited electron is funnelled in the direction of the metabolic pathways of the cells, warranting the conditions for repeated turnover of the RCs. In isolated system in the absence of secondary electron donor and/or acceptors, the RC is reset by the recombination of the “+” and “−” charges. The constitution of the RC and the time constants of charge separation (forward) and recombination (backward) steps are summarized in Fig. 4.

The time constant of the redox cycle of the RC is determined by the redox components bound to it. By changing the appropriate redox component, a wide range of RC turnover rates can be designed (from 10^12^ to 10^−1^ s^−1^). From a practical point of view, it is important to note that every redox cofactor component of the RC can be artificially changed (washed out from the RC and chemically or genetically modified; see e.g. [17, 18]). In general, proteins can be designed and constructed at will, in a broad range, to generate redox processes with different turnover times and conditions.

### The Photosynthetic Membrane

The RC proteins, together with other pigment-protein complexes (light-harvesting complexes; cytochrome bc_1_ or cytochrome bf_6_ (in plants); and the ATP synthase), are embedded in the photosynthetic membrane, which plays central role in the energetics of the cell [19–21]. The energy of light stored in redox potential of the cofactors in the RC (Δ*G* = *n*FΔ*E*_h_) is converted to proton motive force (p.m.f.; [22]), consisting of a transmembrane proton gradient and an electrical potential difference:

$$ \left(\mathrm{p}.\mathrm{m}.\mathrm{f}.=\right)\varDelta {\mu}_{H^{+}}=\frac{\varDelta G}{F}=U-\frac{2.3RT}{F}\varDelta pH $$

Here *n* is the number of moles, Δ*E*_h_ is the redox potential according to the Nernst equation, F is the Faraday constant (=0.0965 kJ/mol mV), R is the universal gas constant, *T* is the absolute temperature, *U* is the membrane potential. The p.m.f. serves the driving force of the ATP synthesis [19, 23].

### RCs in Nanostructures

It is interesting to note that even though the RC proteins—called the “nature’s solar power stations”—have changed the surface of our globe, these are “real nano-systems”. Their size represents only nanometre scale in the cells (about 10 nm [24–26]), and their work is also only “nano” (one photon initiates one charge separation [13]). This protein possesses such technical properties that unique applications are possible, for example, its use in the nanostructures or in the optoelectronic systems [3, 4, 27–29]. These properties prompted huge efforts for creating bio-nanocomposite materials and using RCs attached to different carrier matrices and led to numerous publications. The physico-chemical properties (photochemical activity, electric conductivity) of the bio-nanocomposite materials containing RCs and different carrier matrices have been investigated. A couple of examples will be presented in the following paragraphs.

#### Artificial Lipid Membranes (Proteo-liposomes)

Biological materials are developed by nature for extremely efficient, sensitive and specific functions. When they are isolated from their natural environment, their activity is usually decreased. One obvious possibility is to combine them with nano-systems which mimic the in vivo conditions. For this purpose, artificial lipid membranes (liposomes) seem to be good and obvious choices [30, 31]. On the one hand, RC is a transmembrane protein, so that lipid membrane environment resembles the in vivo conditions [32–35]. In addition, the liposome/RC nanosystem can be a good model for the Mitchell chemiosmotic hypothesis (awarded by Nobel Prize in Chemistry in 1978 [19]), which is the basis of the cell bioenergetics. The radius of the specifically prepared liposome embedding the RCs is about 100 nm [32], which falls in the range of the systems usually called “nano”. Note that this size is about one fourth of the wavelength of the visible light. When the liposome/RC system is illuminated by light, the electromagnetic field is inherently heterogeneous because of the commensurate sizes of the diameter of proteo-liposomes and the wavelength of the visible light.

**Fig. 5:**
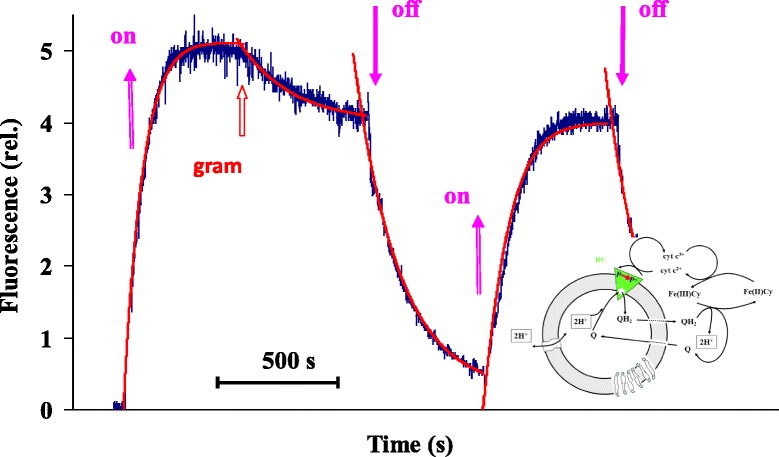
Change of the fluorescence intensity of a pH-sensitive dye (pyranine) during the photocycle of the RC. Fluorescence increase indicates pH increase inside the liposomes. Light is switched on and off, as indicated by the *arrows*. The effect of the protonophore gramicidin (gram) is also shown. Insert: Schematic representation of the RC turnover photocycle. Upon excitation by light P/P^+^//Q/Q- redox pair is created. The P/P^+^ couple is connected to cytochrome c2^+^/ cytochrome c3^+^ turnover on the donor side. The acceptor side is reset by the release of doubly reduced (and protonated) quinone and binding of the oxidized one to the RC. During the whole photocycle DpH is created

By externally added donors (e.g. cytochrome c_2_) and acceptors (ubiquinone, UQ), conditions for the continuous turnover of the photocycle and the possibility of building up p.m.f. can be warranted. The change in the pH can be monitored by pH-sensitive fluorescent dyes which are sensitive to specific ionophores, like gramicidin, which eliminates the transmembrane proton gradient (Fig. 5, [36]).

#### Carbon-Based Materials

Recently, we have shown that RC proteins can be attached to functionalized and non-functionalized, single-walled (SWCNT [37, 38]) and multiwalled (MWCNT [39]) carbon nanotubes (CNTs). In addition to the physical binding, different strategies have been elaborated for the chemical attachment of the RC protein to CNTs ([40]). The successful binding was visualized by EM and AFM measurements, and the functioning of the RC was checked by flash photolysis experiments. We found that (a) RCs can be attached to CNTs effectively, while they largely retain their activity; (b) this binding induces changes in the intra-protein electron transport (stabilization of the charge-separated state); and (c) there is an electronic communication between the SWCNTs and RCs. Circular dichroism (CD) measurements indicate that the binding of the protein to CNT does not alter the excitonic interactions, i.e. there is no substantial change in the RC structure after the binding [24].

**Fig. 6:**
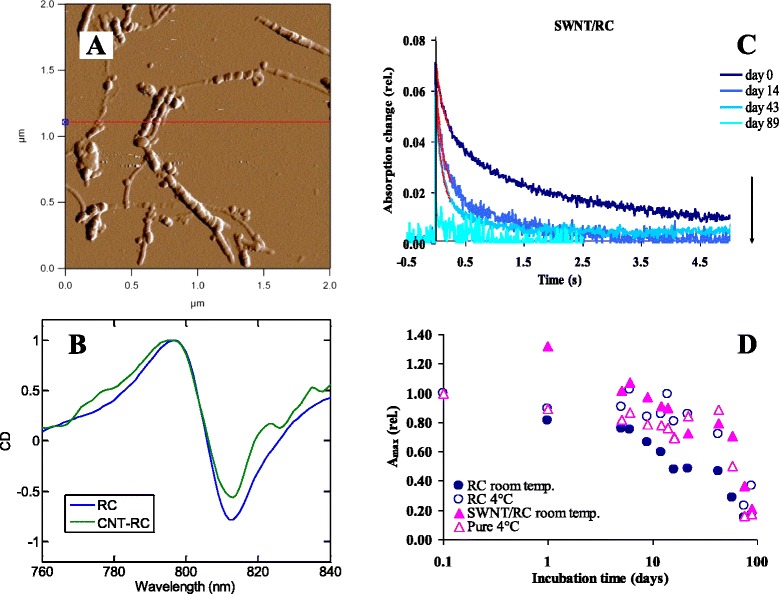
**a** AFM image of the SWCNT/RC complex. RCs are bound to amine-functionalized SWCNTs. **b** Circular dichroism (CD) spectra of RC in detergent solution and bound to SWCNTs as indicated. **c** Absorption change of the SWCNT/RC complex at 450 nm after a single-turnover saturating Xe flash excitation at different incubation times, as indicated. **d** The amplitude of the absorption change of different RC samples as a function of the incubation time. *Filled* and *empty circles* and *triangles* represent RCs and SWCNT/RC complexes at room temperature and at 4 °C, respectively

The long-term stability of the system, which depends on many internal and external factors, is very important for potential applications. Figure 6 shows results of different structural (AFM) and functional (CD, LD, flash photolysis) experiments showing the successful binding of RCs. It is to be noted that after 3 months, the stability drops very suddenly in every sample under the experimental conditions we conducted up to now [38]. However, in the absence of CNTs the inactivation is much faster. There are indirect (via monitoring flash-induced absorption change at specific wavelength or monitoring the redox state of the secondary quinone acceptor by EPR [37]) or direct (measuring the change in the light-induced conductivity of MWCNT bundles/RC composites [40]) proofs of redox interaction between CNTs and the RCs.

#### Transitional Metal Oxide/RC Complexes

Transparent conducting oxides (TCOs) are made of indium oxide (In_2_O_3_), tin oxide (SnO_2_) and ITO, and their combination has been widely used in semiconductor and electronic device industry [41–43]. ITO as a component of the composite electrode in bio-hybrid systems offers unique possibility for application due to its transparency in the visible range. Realization of protein-based bio-nanocomposite materials (among them RC protein-based ones) will lead to conceptual revolution in the development of integrated optical devices, e.g. optical switches, micro-imaging systems, sensors, telecommunications technologies or energy harvesting [44–46].

The spectroscopic properties and electric conductivity of ITO/RC composite provide direct evidence that the RC keeps its photochemical activity even if it is dried on ITO. A direct electric contact between the metal oxide layer and the protein is shown by the light-induced conductivity change of the composite. This bio-nanocomposite provides a model for new generations of applications, e.g. in integrated optoelectronic devices [43].

#### Porous Silicon/RC Complexes

Among the different carrier matrices (carbon-based materials, conductive polymers, different metal oxides like ITO), silicon-based materials have special interest during the engineering of bio-inspired materials [47–56]. Due to its environmental, optical and electronic properties, porous silicon (PSi) is one of the most promising materials not only for biosensors and novel drug delivery systems but also for energy conversion and integrated optoelectronic devices [57–59]. Combination of RC and PSi can offer new possibilities in the field of bio-hybrid systems in nano-bionics.

In an earlier publication, we have shown that the donor and acceptor sites of the RC remained active and accessible for externally added donor (horse heart cytochrome c) and acceptor (UQ-0) agents after binding to PSi. We also predicted a possible redox interaction between the protein and the PSi to be proven directly by an independent experiment [60, 61].

#### Conducting Polymer/RC Complexes

**Fig. 7:**
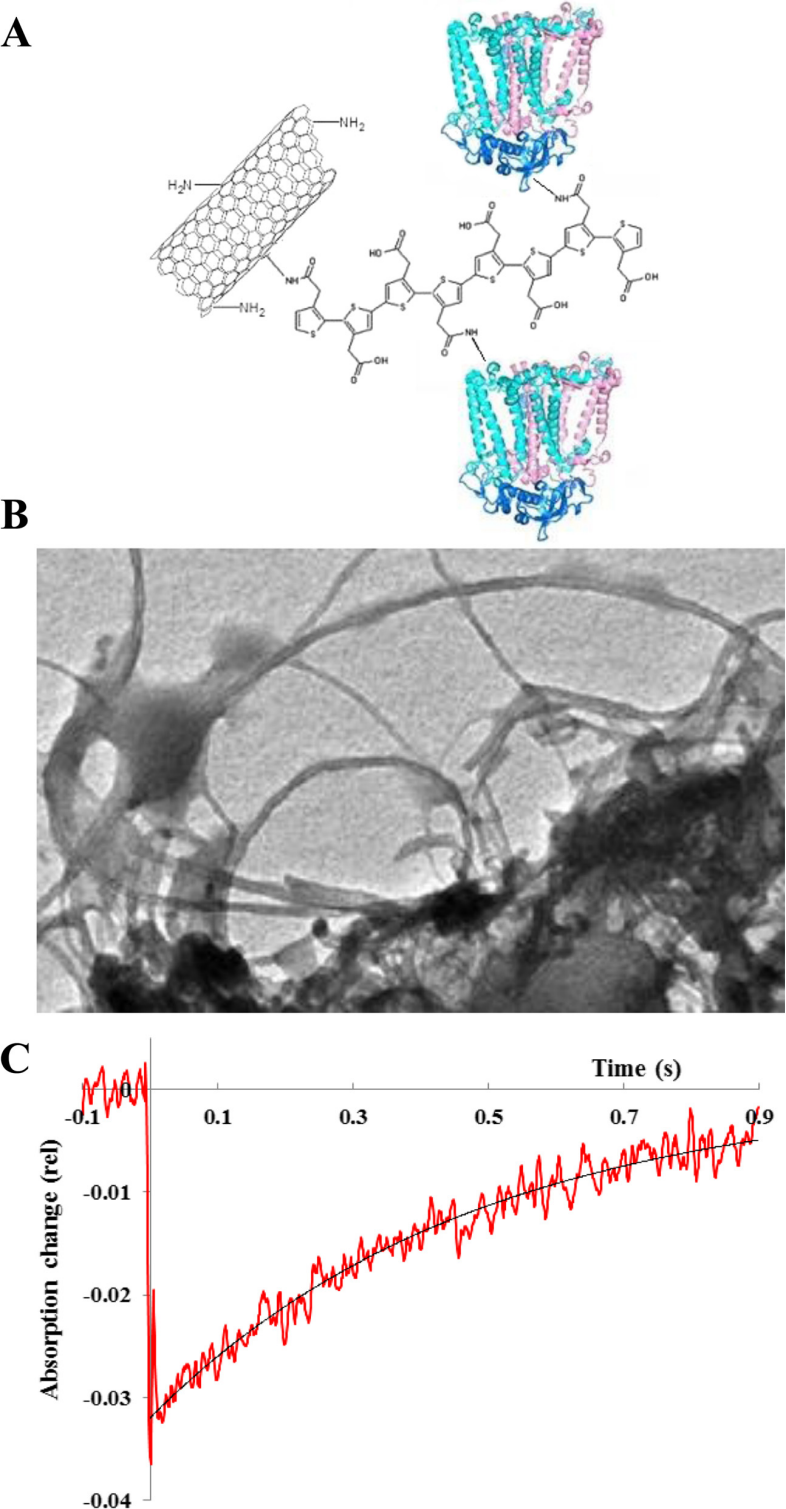
**a** Schematic representation of the MWCNT/PTAA/RC complex. RC: photosynthetic reaction centre; PTAA: poly(3)-thiophene acetic acid conducting polymer; MWCNT: multiwalled carbon nanotube. **b** TEM image and **c** photochemical activity (monitored by the absorption change at 860 nm). The in the absorption at 860 nm monitors the redox state of the primary electron donor (P^+^/P). *Solid line* is calculated by a first-order single-exponential decay with the lifetime of *τ* = 480 ms

The major goal of the current research is to find the most efficient systems and conditions for photoelectric energy conversion and for the stability of the RC bio-nanocomposite. We immobilized the RC protein to MWCNTs through specific chemical binding to amine functional groups and through conducting polymer (poly(3)-thiophene acetic acid, PTAA; Fig. 7). An efficient vectorial charge transfer can be driven in the composites by combining the three systems [62].

The photochemical or photophysical activity of the RC/PTAA/MWCNT complex can be characterized by the absorption change at 860 nm. At this wavelength, the redox state of the primary electron donor bacteriochlorophyll dimer (P^+^/P) can be monitored. The decay curves could be analysed by a multi-exponential fit of sum of first-order reactions: *A*_(t)_ = Σ *A*_(0,*i*)_exp(−*t*/*τ*_(*i*)_), where *A*_(0,*i*)_ and *τ*_(*i*)_ are the contribution and the lifetime of the *i*th component, respectively. However, the monophasic decay, with the lifetime of *τ* = 480 ms, indicates the homogenous functional binding of the RC to the complex. It is expected that the presence of the PTAA increases the electronic coupling between the RC and the MWCNT. In addition, the absorption cross section, which is a determining factor for the power of the energy converter system, is enhanced by the larger local concentration of the RC (i.e. by the larger chromophore density).

### Photocurrent, Optoelectronics

Different biological organizations are used to create new-generation light-harvesting systems. In these composites, the advantages of the biological organizations and the carrier matrices are combined so that these composites can be a state-of-the-art, really smart, modern and innovative device. Photosynthetic RC proteins offer unique applications, for example, their use in the nanostructures or in the optoelectronic systems. In these systems, the electron—arising from charge separation—is trapped in the redox components of the RC or its molecular environment and, among other things, can participate in electric circuits. The fabrication of systems for efficient light energy conversion (e.g. photovoltaics), integrated optoelectronic systems or biosensors (e.g. for specific detection of pesticides) can be visualized for the near future.

**Fig. 8:**
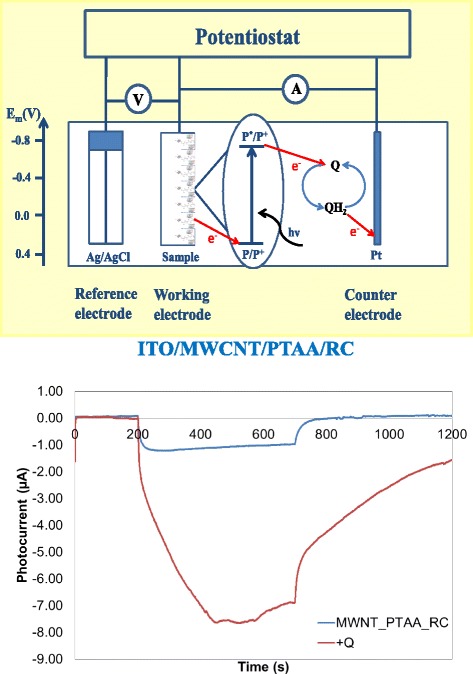
Electrochemical cell (*upper*) and the photocurrent measured by illuminating the ITO/MWCNT/PTAA/RC electrode (*down*). The working electrode is ITO covered by MWCNT/PTAA/RC layer, the counter electrode is Pt, and the reference electrode is Ag/AgCl. The graph shows the light-induced photocurrent of the electrochemical cell without a mediator (blue curve) and with UQ-0 (2,3-dimethoxy-5-methyl-1,4-p-benzoquinone) (*red curve*)

Measuring light-induced change in the current in an electrochemical cell (called photocurrent) is an elegant demonstration of the suitability of the photosynthetic systems for photovoltaics, or other practical applications in optoelectronics (e.g. for sensing elements for specific compounds, like pesticides). Two of our RC-based composites were successfully tested and found to be active in electrochemical cells. It has been demonstrated that continuous redox turnover of nanocomposite prepared from PTAA/MWCNT and RCs bound to ITO can be driven by light if quinone is added to the solution for mediating the electron transport between the working and the counter electrode (Fig. 8).

**Fig. 9:**
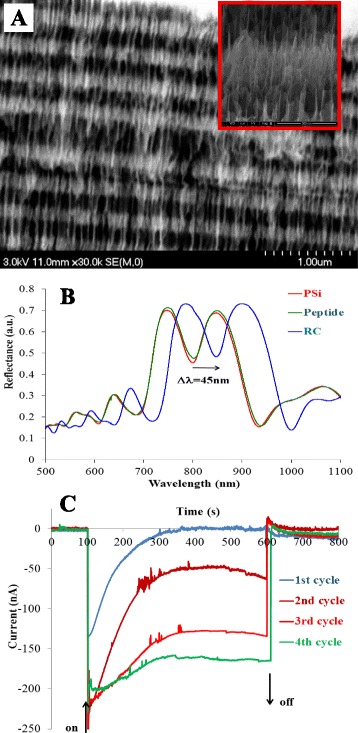
**a** SEM image of the layer structure of PSi. *Insert* shows the three-dimensional arrangement of the microcavity structure. **b** The reflection spectrum of the PSi before and after functionalization with RC. *Arrow* indicates the shift in the specific reflection mode after the RC binding. **c** Light-induced photocurrent with the PSi/RC electrode in the presence of externally added UQ-0 mediator. Traces correspond to the signal during consecutive illumination periods. The measurement was done with the three-electrode electrochemical cell shown in Fig. 7 (Authors are grateful to Dr. G. Palestino (Universidad Autónoma de San Luis Potosí, Mexico) and Dr. Vivechana Agarwal (CIICAP—Universidad Autonoma del Estado de Morelos, Cuernavaca, Mexico) for providing the PSi microcavities for the experiments with RCs.)

Porous silicon/RC complex also shows photocurrent in electrochemical cell indicating that PSi is not only a material for hosting the RC protein in a large surface area but might be an active element in redox transitions as a working electrode (Fig. 9).

### Nano-bionics in Photosynthesis

Nanotechnology offers new and potential direction for using interface between biological (as well as photosynthetic) structures and non-biological nanostructures with enhanced functions for harvesting solar energy or functioning as sensitive photonic chemical sensors [63].

Biological materials and photosynthetic RCs are structured for extremely efficient, sensitive and specific functions. The RC proteins own such technical properties that many unique applications are envisaged, for example, their use in nanostructures or in optoelectronic systems [3, 60]. Different types of RCs found in nature are investigated in different photoconverter applications, and their photoconversion power efficiency is far from the performance in vivo up to now. *η* ≈ 10^−4^ and 10^−7^ are found for bacterial reaction centres in electrochemical cell and dried in semiconductor layer structures, respectively [64]. These values are comparable with the one found for photosystem one (PS-I) in Z-scheme-inspired biophotovoltaics in redox hydrogels (*η* ≈ 10^−5^ [65]).

Although the photoconversion efficiency is very small in these systems up to now, the use of the RCs in bio-nano-systems is a real challenge. This protein assures the energy supply for the whole earthly life (including the fossil fuels as well), and we have this protein in our hand. It can be purified, fully engineered and can be designed to our wish. The function of the RC can be mimicked in many photo-senzibilization and photocatalytic processes in which the charge separation capacity of the protein is substituted by an inorganic analogue.

There is a versatile possibility to construct 2D or even 3D hybrid materials based on graphene and some organic materials in which electron–hole pairs are created upon light irradiation with oxidation and reduction power capability. The special hierarchical semiconductor-based heterogeneous organizations of these organic semiconductor catalysts can mimic the energetic schemes of the photosynthetic RCs which fulfil the conditions of separation and stabilization of light-driven charges by chain of redox reactions [66–68].

Due to its remarkable mechanical, thermal, optical and excellent electron conductivity, high transparency and unique two-dimensional (2D) morphology graphene became a promising material as a component of many optoelectronic assemblies. Coupling with proper semiconductor materials, the exceptional properties of graphene provide the possibility of designing new-generation high performance hierarchically structured artificial photosynthetic systems. The enhanced photochemical activity of these highly structured composites can be utilized in many fields of optoelectronics like environmental remediation, water splitting, CO_2_ photoreduction and selective organic transformation, reviewed, e.g. in [69].

## Conclusion

In this review we have shown that the vectorial charge transfer within the photosynthetic organisms initiated by light is assured in a system organized hierarchically from molecules to molecular complexes. The energy of light which is captured in the form of redox free energy of cofactors can be used for useful work either wiring out the electron in electric circuits or reducing chemical equivalents. This can be a basis of constructing artificial light-energy converting molecular devices like sensing element of bio-hybrid device for biosensor and/or for opto-electronic applications. Future researches should aim to find the most efficient energy converter system (the biological and the inorganic parts), the highest reproducibility and stability of the function.
